# Rationally Designed Interfacial Peptides Are Efficient *In Vitro* Inhibitors of HIV-1 Capsid Assembly with Antiviral Activity

**DOI:** 10.1371/journal.pone.0023877

**Published:** 2011-09-08

**Authors:** Rebeca Bocanegra, María Nevot, Rosa Doménech, Inmaculada López, Olga Abián, Alicia Rodríguez-Huete, Claudio N. Cavasotto, Adrián Velázquez-Campoy, Javier Gómez, Miguel Ángel Martínez, José Luis Neira, Mauricio G. Mateu

**Affiliations:** 1 Centro de Biología Molecular “Severo Ochoa”, Consejo Superior de Investigaciones Científicas-Universidad Autónoma de Madrid, Madrid, Spain; 2 Fundació IrsiCaixa, Hospital Germans Trias i Pujol, Badalona, Barcelona, Spain; 3 Centro de Biología Molecular y Celular, Universidad Miguel Hernández, Elche, Alicante, Spain; 4 Institute for Biocomputation and Physics of Complex Systems, Zaragoza, Spain; 5 Aragon Health Sciences Institute, CIBERed, Zaragoza, Spain; 6 School of Health Information Sciences, The University of Texas Health Science Center at Houston, Texas, United States of America; 7 Fundación ARAID, Diputación General de Aragón, Zaragoza, Spain; Queensland Institute of Medical Research, Australia

## Abstract

Virus capsid assembly constitutes an attractive target for the development of antiviral therapies; a few experimental inhibitors of this process for HIV-1 and other viruses have been identified by screening compounds or by selection from chemical libraries. As a different, novel approach we have undertaken the rational design of peptides that could act as competitive assembly inhibitors by mimicking capsid structural elements involved in intersubunit interfaces. Several discrete interfaces involved in formation of the mature HIV-1 capsid through polymerization of the capsid protein CA were targeted. We had previously designed a peptide, CAC1, that represents CA helix 9 (a major part of the dimerization interface) and binds the CA C-terminal domain in solution. Here we have mapped the binding site of CAC1, and shown that it substantially overlaps with the CA dimerization interface. We have also rationally modified CAC1 to increase its solubility and CA-binding affinity, and designed four additional peptides that represent CA helical segments involved in other CA interfaces. We found that peptides CAC1, its derivative CAC1M, and H8 (representing CA helix 8) were able to efficiently inhibit the *in vitro* assembly of the mature HIV-1 capsid. Cocktails of several peptides, including CAC1 or CAC1M plus H8 or CAI (a previously discovered inhibitor of CA polymerization), or CAC1M+H8+CAI, also abolished capsid assembly, even when every peptide was used at lower, sub-inhibitory doses. To provide a preliminary proof that these designed capsid assembly inhibitors could eventually serve as lead compounds for development of anti-HIV-1 agents, they were transported into cultured cells using a cell-penetrating peptide, and tested for antiviral activity. Peptide cocktails that drastically inhibited capsid assembly *in vitro* were also able to efficiently inhibit HIV-1 infection *ex vivo*. This study validates a novel, entirely rational approach for the design of capsid assembly interfacial inhibitors that show antiviral activity.

## Introduction

Virus morphogenesis involves the self-assembly of the viral capsid through specific interactions between protein subunits, which makes this process an excellent target for antiviral research. In recent years, a number of experimental capsid assembly inhibitors have been identified by screening available compounds, or by selection from chemical or peptide libraries. In addition, the remarkable knowledge acquired on the structure of viruses and the determinants of molecular recognition has made feasible a complementary antiviral approach, based on the rational design of inhibitors able to competitively interfere with interactions between capsid subunits.

Antiviral research has been most active on the development of effective therapies against the human immunodeficiency virus (HIV). Current therapies against HIV involve the use of cocktails of drugs able to inhibit the activity of the viral reverse transcriptase, protease or integrase, or virus entry into cells. Despite the success of this combined approach, the increasing emergence of drug-resistant HIV variants is becoming a serious concern, and demands the development of new anti-HIV agents to be included in future combination therapies (reviewed in ref. [1]). Assembly of the HIV-1 capsid is being actively studied to an extraordinary level of detail (recently reviewed in refs. [2]–[5]), and there is a growing interest in the investigation of new anti-HIV approaches based on the inhibition of capsid assembly.

During HIV-1 morphogenesis, the capsid protein (CA) participates in two distinct assembly events. The first event occurs during formation of an immature, non-infectious virus in the cell, and involves the self-assembly of a spherical capsid comprising up to 5000 molecules of the Gag polyprotein, of which CA constitutes a part. The second event occurs during virus maturation. Gag is proteolytically processed, the immature capsid disassembles, and about 1000–1500 released CA molecules self-associate to form a mature, conical-shaped capsid. The mature capsid is structurally organized essentially as a lattice of CA hexamers. The CA subunits are composed of two domains, the N-terminal domain (NTD) and the C-terminal domain (CTD). Within each hexamer, each CA subunit is connected with another through NTD-NTD interfaces and NTD-CTD interfaces; each hexamer is connected to the six neighboring hexamers through CTD-CTD dimerization interfaces.

Capsid-like particles with the structural organization of mature HIV-1 capsids can be efficiently assembled *in vitro* from free CA molecules under non-physiological, very high ionic strength conditions [6]–[8]. More recently, CA assembly was achieved *in vitro* in close to physiological conditions, including low ionic strength and a very high chemical activity (*effective concentration*) of CA [9]; this was done by adding high concentrations of an inert macromolecule to mimic the macromolecularly crowded environment inside the maturing HIV-1 virion [4], [9]. The availability of simple *in vitro* assembly assays, and the remarkable structural and biochemical properties of CA, provide ample opportunities for the development of inhibitors of HIV-1 capsid assembly.

A few inhibitors of immature or mature HIV-1 capsid assembly have been recently discovered (reviewed in refs. [5], [10]–[13]) by combinatorial approaches based on random libraries of small organic molecules (CAP-1 [14]; PF-3450074 [15], [16], and the dodecapeptide CAI [17]). CAP-1, PF-3450074 and peptide NYAD-1 (a conformationally restricted derivative of CAI [18]), were able to penetrate cultured cells and inhibit HIV-1 infection *ex vivo*. CAP-1 and PF-3450074 bind to different sites in NTD [15], [19]. CAI and its derivative NYAD-1 bind to a hydrophobic pocket in CTD [20]–[22]; CAI not only inhibits mature capsid assembly but also facilitates capsid disassembly [23]. In addition, α-hydroxy glycineamide interfered with HIV-1 morphogenesis [24]–[26], and a dendrimeric compound bound CTD and weakly interfered with CA polymerization *in vitro* [27]. Despite these and other advances using different viral systems [28]–[31], no drugs based on inhibition of capsid assembly have been yet approved for clinical use against HIV-1 or any other virus.

**Figure 1:**
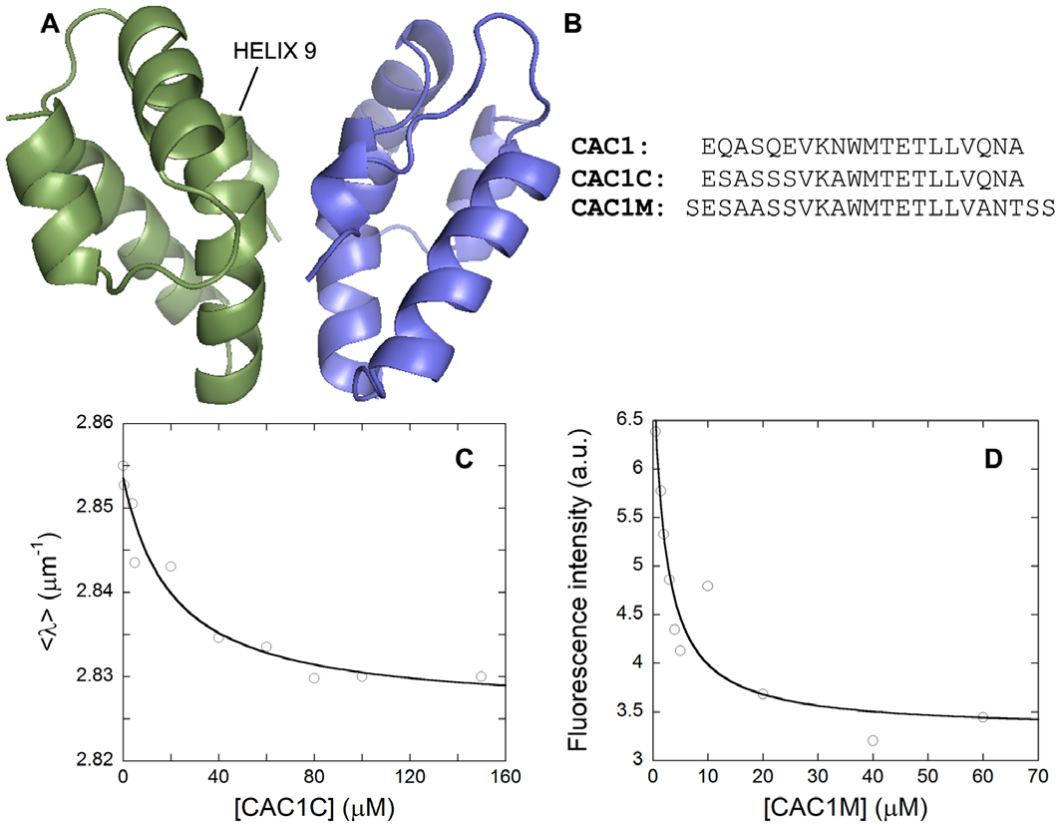
Structure of CTD, and determination of the binding affinity for CTD of CAC1-derived peptides. (A) Ribbon model of the three dimensional structure of the CTD dimer [32]. The two monomers are represented in green or blue. Helix 9 is labelled. (B) Sequence of CAC1 including helix 9 residues, and of designed CAC1-derived peptides. (C) Variation in <λ> of the fluorescence emission spectrum of wild-type dimeric CTD with the concentration of added CAC1C. Fitting to a 1∶1 binding isotherm as described [40] is indicated by a thin line. A similar affinity constant was obtained when the fluorescence intensity at 315 nm instead of <λ> was represented. (D) Variation in fluorescence emission intensity at 315 nm of wild-type dimeric CTD with the concentration of added CAC1M.

As a novel approach, we have undertaken the rational design of peptides that could act as inhibitors of HIV-1 capsid assembly by mimicking capsid structural elements involved in intersubunit interfaces to competitively inhibit CA oligomerization. The first targeted interface has been the CTD-CTD dimerization interface. Its structural description in the dimer in solution [32], [33]; (Fig. 1A) is consistent with descriptions as a part of the mature HIV-1 capsid [34]–[38]. This interface is essentially formed by the parallel packing of helix 9 (Fig. 1A), with the participation of interactions between residues in the 3_10_ helix of a monomer and residues in helices 9 and 10 of the other monomer. As the isolated CTD dimerizes with the same affinity as full-length CA [32], it likely includes all the energetically significant CTD-CTD interactions present in the mature capsid. This has allowed a detailed quantitative thermodynamic dissection of this interface [39].

The structural and thermodynamic data on the CTD dimerization interface summarized above led us to the design of a 20-mer peptide (CAC1) that represents the sequence of helix 9, and includes most of the energetically important residues at the CTD dimerization interface. We showed then that CAC1 is able to bind CTD with an affinity only 5-fold lower than the dimerization affinity of the complete CTD [40].

In the present study we have i) rationally designed variants of the CAC1 peptide to increase its solubility and CTD binding affinity; ii) mapped the binding site in CTD of CAC1 and CAC1-derived peptides; iii) designed four additional peptides that represent other CA helical segments also involved in mature capsid assembly; iv) tested the inhibitory activity of the designed peptides, alone or in combination, on the *in vitro* assembly of the mature HIV-1 capsid; and v) transported the peptides into HIV-1-susceptible cells using a heterologous cell-penetrating peptide, and analyzed the inhibitory activity of the peptides, alone or in combination, on HIV-1 infection in cultured cells. The results validate an entirely rational approach for the design of interfacial inhibitors of virus capsid assembly *in vitro* that show antiviral activity *ex vivo*.

## Materials and Methods

### Synthetic peptides

CAC1 (E^175^QASQEVKNWMTETLLVQNA^194^) [40], CAC1-derived peptides CAC1C (E^175^SASSSVKAWMTETLLVQNA^194^),CAC1M(SE^175^SAASSVKAWMTETLLVAN^193^TSS) and a control, non CTD-binding, thioredoxin binding peptide (ESLPDYGDDSDDNTLGTEEQRSIVNVSQ) derived from pea chloroplast fructose-1,6-biphosphatase (Fbase), were synthesized and purified by Genscript, NJ, USA. Peptides H2 (K^30^AFSPEVIPMFSALSEGAT^48^), H3 (Y^40^SALSEGATPQDLfNTMLNT^58^), H4 (H^62^QAAMQMLKETINEEAAEWDRVH^84^), H8 (K^158^EPFRDYVDRFYKTLRAEQ^176^), CAI (ITFEDLLDYYGP” [17]), and also some batches of CAC1 and CAC1M, were synthesized and purified by the Peptide Synthesis Facility, Universitat Pompeu-Fabra, Barcelona, Spain. N-terminus fluorescein (FITC)-labelled CAI and rhodamine-labelled peptides CAI, CAC1M and H2 were obtained from the latter source. All peptides were amidated at the C-terminus. Numbering refers to amino acid position in the CA protein sequence. The Chariot peptide transfection reagent was from Active Motif.

### Biological materials

NB324K cells (kindly provided by Prof. J.M. Almendral) were used to set up the peptide transport protocol. NB324K cells were cultured in Dulbecco's modified Eagle's medium (DMEM, Gibco) supplemented with 5% fetal bovine serum (FBS, Gibco), 2 mM L-glutamine, 75 U/ml streptomycin and 75 µg/ml penicillin G, at 37°C in a 5% CO_2_ atmosphere. Human astroglioma cell lines (U87-CD4-CXCR4; AIDS Research and Reference Reagent Program, National Institutes of Health (NIH), USA; catalog number 4036) expressing CD4 and CXCR4 receptors were used for peptide transport and HIV-1-production-infection inhibition assays. U87-CD4-CXCR4 cells were cultured in DMEM supplemented with 10% FBS, 0.5 mg/ml geneticin and 1 µg/ml puromycin, at 37°C, in a 5% CO_2_ atmosphere. For infection of U87-CD4-CXCR4 cells the HXB2 strain of HIV-1 was used.

### Protein expression and purification

The ^15^N-labelled, monomeric CTD mutant W184A was obtained by using Bioexpress medium (Cambridge Isotope Laboratories, MA, USA). CA protein and the isolated CTD (either unlabelled or labelled) were purified as described previously [39]. Protein stocks were run in SDS-PAGE gels and found to be essentially pure. Protein concentration was determined as described [41].

### Fluorescence spectroscopy

Spectra were collected on a Cary Eclipse spectrofluorimeter (Varian, CA, USA). A concentration of 20 µM CTD and increasing peptide concentrations were used for titration experiments carried out at 25°C in 50 mM phosphate buffer pH 7.0. Excitation wavelength was 280 nm or 295 nm, and emission fluorescence was collected between 300 nm and 400 nm. The excitation and emission slits were set to 5 nm. The response time was 1 s. The dissociation constant of each complex was calculated by fitting the changes observed either in: (i) the fluorescence intensity at a particular wavelength; or (ii) the value of the average energy <λ> [42] for a fixed CTD concentration *versus* the concentration of added peptide as described [40].

### Circular dichroism (CD) spectroscopy

Spectra were collected at 25°C in 50 mM phosphate buffer pH 7.0 using a J810 spectopolarimeter (Jasco, Japan). Far-UV measurements were performed using equimolar amounts of CTD and the corresponding peptide (20 µM or 200 µM) in 0.1 cm-pathlength quartz cells (Hellma). Molar ellipticities, [Θ], were calculated as described [40].

#### (a) Steady state CD measurements

Spectra were acquired with a response time of 2 s, and averaged over 6 scans, with a scan speed of 50 nm/min. The step resolution was 0.2 nm, and the band-width was 1 nm. Experiments aimed at analysing the self-association of the peptides and estimation of the dissociation constant were carried out as described previously [40].

#### (b) Thermal-denaturation CD analysis

The ellipticity at 222 nm was followed using a rate of 60°C/h, and a response time of 8 s. The [protein]/[peptide] concentrations used were 20/20 µM, 20/40 µM, 20/60 µM or 200/200 µM. Fitting of the sigmoidal curves was carried out by using the program Kaleidagraph (Abelbeck Software).

### Isothermal titration calorimetry (ITC)

ITC measurements were performed by using a VP-ITC isothermal titration calorimeter (MicroCal, MA, USA). Measurements were carried out at 25°C in 12 mM Tris or phosphate buffer pH 7.0. The sample cell (1.4 ml) was loaded with CTD at 20 µM; the corresponding peptide was loaded into the syringe at a concentration of 400 µM. Twenty-eight injections of 10 µl were added sequentially to the sample cell every 400 s to ensure that the reaction was completed (as detected by the calorimetric signal reaching the baseline level before the next injection). The amount of power required to maintain the reaction cell at constant temperature after each injection was monitored as a function of time. The isotherms (differential heat upon binding *versus* the molar ratio: [peptide]/[CTD]) were fitted to a single-site model assuming a 1∶1 stoichiometry for the CTD∶peptide complex. Data were analyzed with software package Origin 7.0 (OriginLab) provided by the instrument supplier. As a control experiment, the individual dilution heats for the isolated peptides were determined under the same experimental conditions described above, by carrying out identical injections of the peptide into the sample cell, which contained only buffer.

### NMR spectroscopy

NMR spectra were acquired at 20°C on a Bruker Avance DRX-500 spectrometer (Bruker GmbH, Germany) equipped with a triple resonance probe and z-pulse field gradients. Sodium trimethylsilyl [2,2,3,3-^2^H_4_] propionate (Sigma) was used as the internal chemical shift reference in the homonuclear experiments. All experiments were carried out in 50 mM phosphate buffer pH 7.0. Assignments of the heteronuclear single quantum coherence (HSQC) NMR spectra were those previously obtained for the monomeric CTD [43], [44].

#### (a) 2D ^15^N-^1^H-HSQC-NMR spectra

All spectra were acquired in the phase-sensitive mode. Frequency discrimination in the indirect dimensions was achieved by using the States-TPPI method [45]. The 2D-^15^N-HSQC experiments [46] were acquired with 4 K data points in the ^1^H dimension and 200 scans were acquired in the ^15^N axis. The spectral width was 15 ppm in the ^1^H dimension and 35 ppm in the indirect ^15^N one. Water was suppressed with the WATERGATE sequence [47]. The concentration of CTD W184A was in all cases 120 µM, and the corresponding peptide concentration was four-fold higher. To identify CTD residues potentially involved in peptide binding, changes in normalized peak intensity were taken into account, as significant changes in chemical shifts are not observed when different compounds are specifically bound to this protein [27], and for other protein-ligand complexes (e.g. ref. [48]). Changes in intensity were small but entirely reproducible and were quantitated by determining the intensity of the signals in the HSQC spectra and normalizing them with respect to the signal of Leu231 (the CTD C-terminal residue); in that way, errors in protein concentration and/or in the comparison between two spectra acquired at different days with two samples were avoided. Only those residues whose normalized intensities in the absence and in the presence of CAC1C or CAC1M showed differences above a threshold of 10 units (Table S1) were considered to be affected by binding. As an example, the row corresponding to Arg154 in CTDW184A, in the absence or presence of CAC1C, is shown in Fig. S1. We also acquired an HSQC spectrum in the presence of the Fbase peptide with CTDW184A at a 2∶1 peptide∶protein molar ratio.

#### (b) Measurement of T_2_ (transverse relaxation time)

We measured *T*_2_ of the mutant peptides, and as a control that of CAC1, by using the 1-1 echo sequence (at the echo-times of 2.9 ms and 400 µs) [49]. Then, since the correlation time, τ_c_, is τ_c_≈1/*T_2_* [50], the molecular mass *M* of the biomolecule, about twice the τ_c_, can be estimated. We measured the intensity and the width of Gly156 (the most down-field shifted signal). Given the errors inherent to the technique [50], and the *M* of the peptides, we estimated that variations larger than 10% in the measured *T*_2_ are significant. Peptide concentrations were in the range of 50 µM or 200–300 µM for CAC1 or CAC1A/CAC1M, respectively.

#### (c) Translational diffusion measurements (DOSY experiments)

Translational self-diffusion measurements were performed with the pulsed-gradient spin-echo sequence to determine the aggregation state of the peptides. Measurements were carried out as described [51]. The duration of the gradient was 3 ms, and the time between both gradients was 150 ms. The most up-field shifted methyl groups of the peptides (those between 0.5 and 1 ppm) were used to calculate intensities. Peptide concentrations were in the range of 50 µM or 200–300 µM for CAC1 or CAC1A/CAC1M, respectively.

To determine the *D* of each peptide and, from this value, its corresponding hydrodynamic radius (*R*_h_), we used a previously developed approach [52]. The *R*_h_ of dioxan was 2.12 Å, and its experimentally determined *D*, under our conditions, was 8.17×10^−6^ cm^2^ s^−1^. The gradient strength was calibrated by using the *D* for the residual proton water line in a sample containing 100% D_2_O in a 5 mm tube, as described [51].

### Kinetic analysis of CA polymerization *in vitro*

Assembly reactions were carried out as described previously [9], [53] with some modifications. In general, the polymerization mixtures contained 50 mM sodium phosphate buffer pH 7.4, 2.25 M NaCl, 20 µM CA, and 100 g/l Ficoll-70 (GE HealthCare) as a macromolecular crowding agent. Where indicated, Ficoll-70 was omitted and the final CA concentration was raised to 46 µM. The experiments were initiated by introducing an appropriate volume of a CA solution in 50 mM phosphate buffer into a spectrophotometer cuvette (10-mm pathlength, 20 mm^2^ internal section), and the assembly reaction was triggered by adding the appropriate volume of a solution containing 4 M NaCl, 179 g/l Ficoll-70 in 50 mM sodium phosphate buffer pH 7.4, to get the final concentrations desired for each component in a final volume of 500 µl, followed by rapid mixing by repeated inversion of the cell. The pH of the final reaction mixture was checked in a test sample. For inhibition assays, CA was mixed with the appropriate amounts of the peptides, and incubated for 30 minutes before triggering the reaction as indicated above. The time-dependent increase in the optical density at 350 nm as a measure of the light scattered by the assembled particles was monitored at 25°C using a UV-1603 spectrophotometer (Shimadzu, Japan), with data points collected every six seconds. Traces of the variation in the turbidity were analyzed as described previously [9], [53].

### Transport of peptides into cells and fluorescence microscopy

To transport the peptides into NB324K or U87-CD4-CXCR4 cells a commercial cell-penetrating peptide (Chariot, Active Motif) was used, according to the manufacturer's recommendations. For fluorescence microscopy analyses, Chariot-peptide binding was accomplished by incubating a mixture containing between 5 µg and 50 µg of either FITC-labelled peptide or rhodamine-labelled peptide and the Chariot peptide at a 1∶1 molar ratio in 100 µl aliquots. Each 100 µl aliquot of the preincubated mixture was added to one well of a 24-well plate containing NB324K or U87-CD4-CXCR4 seeded the previous day (5·10^4^ and 1.5·10^5^ cells/well, respectively) and 100 µl of serum-free DMEM medium were added. The cells were incubated for 1–3 h, 800 µl DMEM containing 10% FBS were added, and incubation was continued for 1 h. Cells were fixed with a 1∶1 methanol∶acetone solution, their nucleus were stained with 4′,6-diamidino-2-phenylindol dihydrochloride (DAPI, Calbiochem), and cells were examined and imaged using an Axiovert200 (Zeiss) fluorescence microscope and/or a LSM-510 (Zeiss) confocal fluorescence microscope.

### Flow cytometry analysis

FITC-labelled peptides were transported into NB324K or U87-CD4-CXCR4 cells as described above. Then, the cells were washed three times with phosphate-buffered saline (PBS) and incubated 15 minutes at 37°C with 0.25% (w/v) trypsin to remove the FITC-peptide that could remain attached to the cell membrane. Trypsin was inactivated by adding DMEM/10% FBS, and the cells were pelleted and resuspended in PBS / 1% bovine serum albumin (BSA) / 1% FBS. Propidium iodide (Sigma-Aldrich) was added to a final concentration of 1 µg/ml to label dead cells. The resuspended cells were analyzed in a FACSCalibur flow cytometer (Becton Dickinson, USA).

### Cytotoxicity assays

The peptides were evaluated for toxicity when transported into cells by using a modified tetrazolium-based colorimetric method (MTT assay) [54]. Briefly, cells treated with the peptides were incubated for 72 h, treated with a solution of 3-(4,5-dimethylthiazol-2-yl)-2,5-diphenyltetrazolium bromide (MTT, Sigma-Aldrich) at 7.5 mg/ml, and incubated for 2 h at 37°C. Then, an acidified isopropanol solution was added, and the absorbance at 550 nm and 620 nm was determined.

### Peptide inhibition of HIV-1 production and infection in cultured cells

U87-CD4-CXCR4 cells from cultured monolayers were collected using 0.25% trypsin and resuspended in DMEM supplemented with 10% FBS (without antibiotics). Cells were seeded in 96-well plates at a density of 3.5×10^4^ cells/well and incubated for 24 h. Then, the cells were incubated as described above with mixtures of the corresponding peptides and the Chariot peptide at the appropriate concentrations. After three hours of incubation, the cells were washed with PBS and infected with HIV-1 at a multiplicity of infection of 0.0015 (0.0015 TCID_50_/cell). After incubation for 2 h at 37°C, the cells were washed with PBS and DMEM supplemented with 10% FBS, 0.5 mg/ml geneticin and 1 µg/ml puromycin were added, and the infected cells were further incubated at 37°C. Viral replication was quantified by measuring HIV-1 CA (p24 antigen) production in the culture supernatant 72 h after infection, using an enzyme-linked immunoassay (INNOTEST HIV-antigen, Innogenetics).

### Analyses of structural models

Personal computers, a Silicon Graphics workstation and the programs *RasMol* [55], *Pymol* [56] and *Whatif* [57] were used.

## Results

### Design of CAC1-derived peptides and analyses of their solubility and CTD-binding affinity

Peptide CAC1 represents the sequence of helix 9 in CA (Figs. 1A and 1B) and proved able to bind CTD, but it also showed a tendency to aggregate [40]. We found that CAC1 includes a region (CA residues 176 to 184) with a high propensity for amyloid fibril formation, according to the ZipperDB database [58]; this prediction is in qualitative agreement with that of the AMYPbd database [59]. We hypothesized that breaking amyloid-like signatures by carefully chosen amino acid substitutions could decrease the tendency of CAC1 to aggregate. However, the substituted peptide should preserve the helix 9 residues we had found to be energetically critical for CTD-CTD dimerization [39].

This line of reasoning led to the design of a first CAC1-derived peptide, CAC1C (Fig. 1B) in which Q176, Q179 and E180 were replaced by serine and N183 by alanine. Based on CAC1C, a second variant peptide, CAC1M (Fig. 1B) was designed by: i) replacing S178 by alanine to improve helix propensity, and also because this substitution in CTD led to an increase in dimerization affinity; ii) replacing Q192 by alanine to further increase binding affinity, as this substitution in CTD increased dimerization affinity by over one order of magnitude [39]; and iii) adding further serine residues at both termini (and replacing A194 by threonine) to increase solubility, and to compensate for loss of hydrophilicity caused by some of the other substitutions.

We first analyzed whether the CAC1-derived peptides CAC1C and CAC1M had any tendency to self-associate. For both peptides, CD measurements showed a variation in molar ellipticity at 222 nm with increasing peptide concentrations (Fig. S2); in addition, NMR experiments showed that the diffusion coefficient and the molecular mass calculated from the relaxation measurements were higher than expected for a monomeric species (Table 1). The hydrodynamic radius (*R_h_*) of the three peptides was similar (Table 1). The apparent dissociation constant for the self-association process determined by steady-state CD was comparable for CAC1C, CAC1M and the unmodified CAC1 peptide (Table 1). Together, the above results indicate that both peptides, like its parent CAC1, still had a tendency to aggregate. However, both variant peptides did show an increased solubility compared to CAC1. In the conditions used for NMR experiments, which involved long incubations at a relatively high temperature, CAC1 concentrations higher than 50 µM led to precipitation [40], while CAC1C and CAC1M remained fully soluble even at 200 µM.

**Table 1:** Parameters for self-association and CTD binding of CAC1-derived peptides^a^.

Peptide	M_r_ b (kDa)	Self-association *K*_D_ (µM)	*R_h_* c (Å)	*T*_2_-relaxation time (ms) and (M_r_ d) (kDa)	CTD-binding *K*_D_ (µM)
CAC1	2.319	9±3^e^	7.7	53 (7.5)	50±30^e^
CAC1C	2.152	3.1±0.5	6.9	40 (9.8)	19±8
CAC1M	2.499	3.2±0.6	8.9	80 (5.3)	8±1

a All measurements were carried out at 25°C, except for the determination of the *R*_h_, which was at 20°C.

b Molecular mass for the peptide monomer calculated from its amino acid sequence.

c Hydrodynamic radius of the peptide.

d Molecular mass for the peptide calculated from the relaxation measurements.

e Data taken from [40], and shown here for completeness.

We used next several biophysical techniques to analyze whether CAC1C and CAC1M are able to bind CTD. Thermal denaturation of CTD followed by CD in the absence or presence of different amounts of either peptide was used first. Binding of the peptide to the native state of CTD, in the absence of binding to the denatured state, is expected to lead to an increase in the melting temperature (*T_m_*) of the protein [60], [61]. Analysis of the data obtained by far-UV CD showed that the shape and/or the *T_m_* of CTD changed upon addition of CAC1C or CAC1M (Fig. S3). Furthermore, irreversibility was observed in the presence of the peptides. These results provided evidence of an interaction between the peptides and CTD, and suggest that the peptides may interact not only with the folded state but also, to a certain extent, with the unfolded state of CTD.

We then attempted to measure the binding affinity of CAC1C and CAC1M for CTD by using ITC. This technique yielded a single binding isotherm with CAC1 [40]; however, it did not yield a clear result with the CAC1-derived peptides (Fig. S4) although these peptides did interact with CTD, as demonstrated by using several other techniques (see below). One possibility to explain the ITC results is that the CTD-peptide interaction could involve a small global apparent enthalpy; in addition, the absence of a significant buffer ionization enthalpy effect on the interaction may indicate that there is no net proton exchange with the bulk solution upon CTD-peptide complex formation.

As an alternative to ITC, we used spectrofluorimetry to determine the binding affinity for CTD of the CAC1-derived peptides (Figs. 1C and 1D). For the two peptides, CTD fluorescence decreased upon addition of increasing concentrations of either peptide, suggesting interactions with either the sole tryptophan of the protein (W184, centrally located at the dimerization interface), or with its neighborhood. Fitting of the variation in fluorescence intensity or <λ> yielded an apparent equilibrium dissociation constant *K_d_* that, for CAC1C and especially CAC1M, was smaller than the *K_d_* determined for CAC1 using the same approach [40] (Table 1). Thus, the rational modification of CAC1 led to peptides that showed a higher solubility, and a moderately increased affinity for CTD.

### Mapping the binding site in CTD of CAC1 and CAC1-derived peptides

To locate the binding sites of CAC1 and CAC1-derived peptides on the CTD structure we carried out 2D ^15^N-^1^H-HSQC spectroscopic measurements of the monomeric CTD mutant W184A in the absence or presence of the peptides. The monomeric mutant closely mimics the conformation of a monomeric intermediate during (non-mutated) CTD dimerization [43], [62]. We used monomeric CTD instead of dimeric CTD to facilitate i) the assignment of resonances by precluding severe signal broadening arising from the dimerization equilibrium [22], [43], [63], and ii) comparison with the results of previous analyses that used the same approach to map the binding site in CTD of the CAI peptide and its derivative NYAD-1 [16], [17], [22].

**Figure 2:**
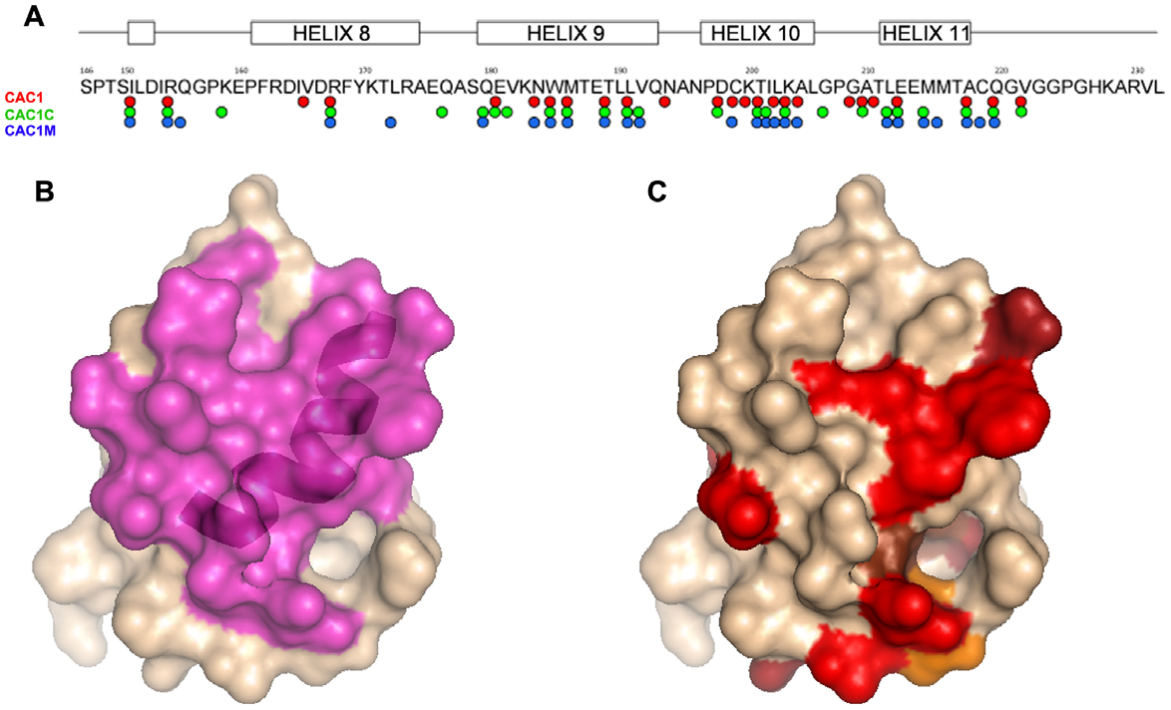
Mapping by NMR spectroscopy of the binding site in CTD of CAC1 and CAC1-derived peptides. (A) Mapping on the CTD sequence of the residues affected by peptide binding. Red, green or blue circles denote residues affected by binding of CAC1, CAC1C or CAC1M, respectively. (B, C) Surface model of one of the monomeric subunits in the CTD dimer. In (B) the residues involved in the CTD dimerization interface are coloured magenta. The ribbon tracing of the helix 9 main chain is superimposed. In (C) the residues affected by binding of the peptides are colored as follows: bright red, residues affected by binding of any peptide; dark red, orange or brown, residues affected by binding of CAC1 and CAC1C, CAC1 and CAC1M, or CAC1C and CAC1M, respectively.

The results obtained (Table S1 and Fig. 2) were consistent with what was expected from peptides that mimicked helix 9: most CTD residues whose signals in the HSQC spectra showed the largest variations on peptide binding were at or close to the CTD dimerization interface and overlapped substantially with the position for helix 9 in the CTD dimer (compare Figs. 2B and 2C). Also as expected, the binding sites of CAC1 and related peptides overlapped extensively (Figs. 2A and 2C). In particular, residues 184, 185, 188, located at the central part of CA helix 9, and residues 150, 154, 190, 200 and 203 are at, or close to, the binding sites of all three peptides and the CTD dimerization interface. In the presence of shorter mutant CAC1 peptides, CTD yielded HSQC spectra in which the residues whose signal intensities changed were different from those observed in the presence of CAC1, CAC1C and CAC1M. This indicated that the shortened mutant peptides bind at a different CTD region than CAC1, consistent with the lack of inhibitory activity on CA polymerization of the shortened peptides (R.D., R.B., M.G.M. and J.L.N., unpublished results). As expected, in the presence of a Fbase peptide, that was unable to bind CTD, no variation in signal intensity for any CTD residue was observed. These results provided an additional control to the specificity and approximate definition of the binding sites identified in CTD for CAC1, CAC1C and CAC1M. The HSQC mapping results are also entirely consistent with the inhibition of CTD dimerization by CAC1 [40] and with the activity and specificity of CAC1 and derived peptides as inhibitors of capsid assembly (see next).

### Inhibition of the *in vitro* assembly of HIV-1 capsid-like particles by CAC1 and CAC1-derived interfacial peptides

The putative inhibitory activity of peptide CAC1 on HIV-1 capsid assembly had not been previously tested. To analyze this possibility, we have used an *in vitro* assay that allows CA polymerization into capsid-like particles [6]–[8]. In order to increase the chemical activity of CA using low protein concentrations, a macromolecular crowding agent (Ficoll-70) was added to the CA polymerization mixtures [9], [53]. We have repeatedly confirmed by electron microscopy that the increase in turbidity observed on CA polymerization corresponds to the formation of tubular structures with the basic structural organization of authentic mature HIV-1 capsid-like structures ([9] and Fig. S5), as previously determined by different authors using the same procedure in various conditions [6]–[8], [34].

**Figure 3:**
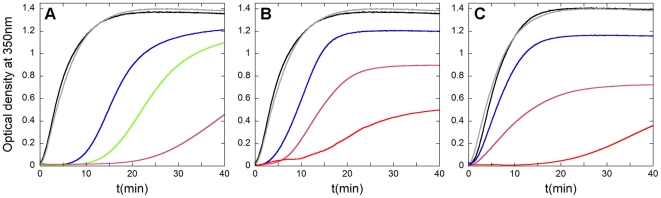
*In vitro* inhibition of HIV-1 capsid assembly by CAC1 and CAC1-derived peptides. Reaction kinetics of CA polymerization was followed by measurement of the optical density at 350 nm as a function of time after triggering the reaction. (A) Inhibition by free full-length CTD, used as a reference inhibitor. (B) Inhibition by CAC1. (C) Inhibition by CAC1M. The CA concentration was 20 µM, and 100 g/l of Ficoll-70 as an inert crowding agent was used. The traces are colored as follows: no free CTD or peptides added (black); an inactive peptide added at a peptide∶CA molar ratio of 10, added as a negative control (grey); added CTD (A), CAC1 (B) or CAC1M (C) at inhibitor∶CA molar ratios of 1 (blue), 2 (green), 5 (pink) or 10 (red).

The isolated CTD was used here as a positive control for efficient inhibition of HIV-1 capsid assembly *in vitro* (Fig. 3A). A peptide unable to bind CA did not show any inhibitory activity, even at a very high peptide∶CA molar ratio (Fig. 3B). In contrast, CAC1 was able to inhibit capsid assembly with an efficiency that approached that of the complete CTD (Fig. 3B). This observation is in full agreement with the results described above on binding affinity for CTD and the extensive overlapping between the CAC1 binding site and the CTD dimerization interface. CAC1C was able to inhibit capsid assembly with an efficiency that was not significantly higher than that of CAC1. However, CAC1M did reproducibly show a moderately increased inhibitory activity compared to CAC1 (about 20% higher reduction in total capsid production, 30% higher reduction in polymerization rate at a peptide∶CA molar ratio of 5) (Fig. 3C); this result is consistent with the somewhat increased binding affinity for CTD of the CAC1M variant peptide compared to CAC1 (Table 1).

By excluding the crowding agent, the CA polymerization rate and the amount of capsid formed were drastically reduced, as expected from theoretical considerations on macromolecular crowding and our previous experimental work [9], [53]. However, by substantially increasing CA concentration, efficient assembly was restored. Testing in these conditions the inhibitory activity of peptides CAC1, CAC1M and other peptides led to results (Fig. S6) that were qualitatively similar to those obtained in the presence of a crowding agent.

### Design of other interfacial peptides that mimic the sequence of different helical regions of CA involved in mature HIV-1 capsid assembly, and *in vitro* analysis of their inhibitory activity on capsid assembly

The results described above showed that the rationally designed CAC1 peptide and, especially, its improved variant CAC1M closely approach *in vitro* the inhibitory activity of the complete CTD domain on HIV-1 capsid assembly. Thus, we decided to attempt the design of further inhibitors of HIV-1 capsid assembly by targetting additional CA oligomerization interfaces, using an approach similar to that followed for CAC1 [40].

**Figure 4:**
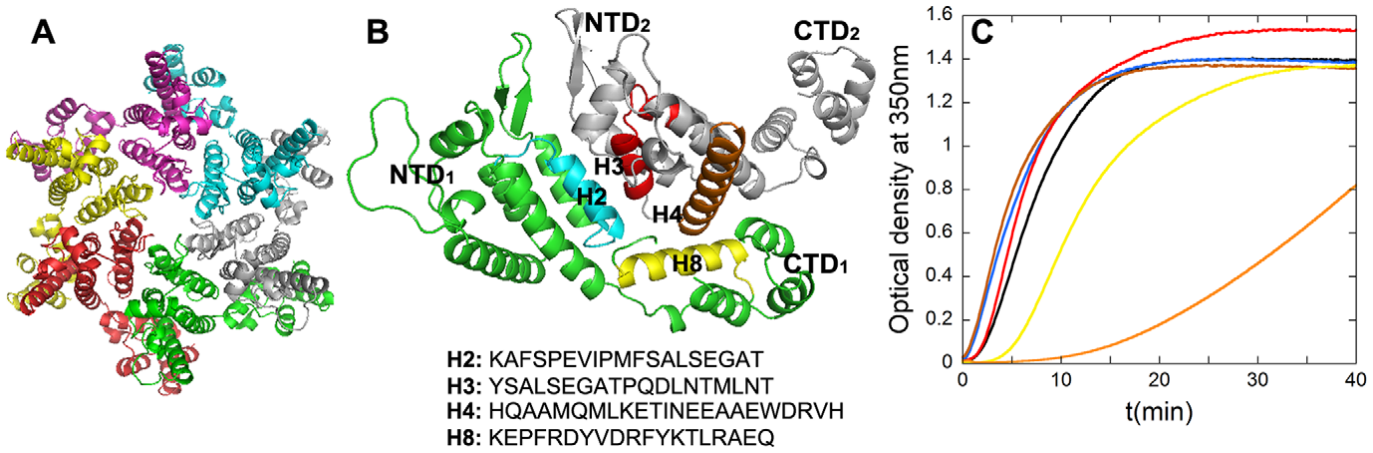
Inhibition of *in vitro* capsid assembly by peptides H2, H3, H4 and H8. (A) Crystallographic structural model of the CA hexamer [64]. Each CA monomer is shown in a different color. (B) Closeup of the arrangement of two neighboring CA monomers (labelled 1 and 2) in the CA hexamer depicted in panel (A). The orientation has been modified to show the CTD-NTD and NTD-NTD interfaces more clearly. Helices 2 in blue, 3 in red, 4 in orange and 8 in yellow are highlighted, and labelled H2, H3, H4 and H8, respectively. The sequences of the corresponding peptides are indicated. (C) Reaction kinetics of CA polymerization using the conditions described in the legend for Fig. 3. The traces are colored as follows: no peptide added (black); peptide H2 (blue); peptide H3 (red); peptide H4 (brown), all at a peptide∶inhibitor molar ratio of 10; peptide H8 at a peptide∶CA ratio of 5 (yellow); peptide H8 at a peptide∶CA molar ratio of 10 (orange).

CA helices 2 and 3 in NTD are critically involved in the NTD-NTD interfaces and CA hexamerization; CA helices 4 in NTD and 8 in CTD are critically involved in NTD-CTD heterodimerization within each CA hexamer [64] (Figs. 4A and 4B). Thus, synthetic peptides that represent the sequences of those four helical segments were designed (peptides H2, H3, H4 and H8 respectively; Fig. 4B), and tested *in vitro* for activity as potential interfacial inhibitors of mature HIV-1capsid assembly, either in the presence (Fig. 4C) or absence of a macromolecular crowding agent.

Peptides H2, H3 and H4 were not able to inhibit the *in vitro* assembly of HIV-1 capsid-like particles, even at the highest peptide∶CA molar ratios tested. In contrast, peptide H8 did substantially and reproducibly inhibit capsid assembly, although its inhibitory activity was lower than that of CAC1 and CAC1M (Fig. 4C).

### Inhibition of the *in vitro* assembly of the HIV-1 capsid by cocktails of CA-binding peptides

**Figure 5:**
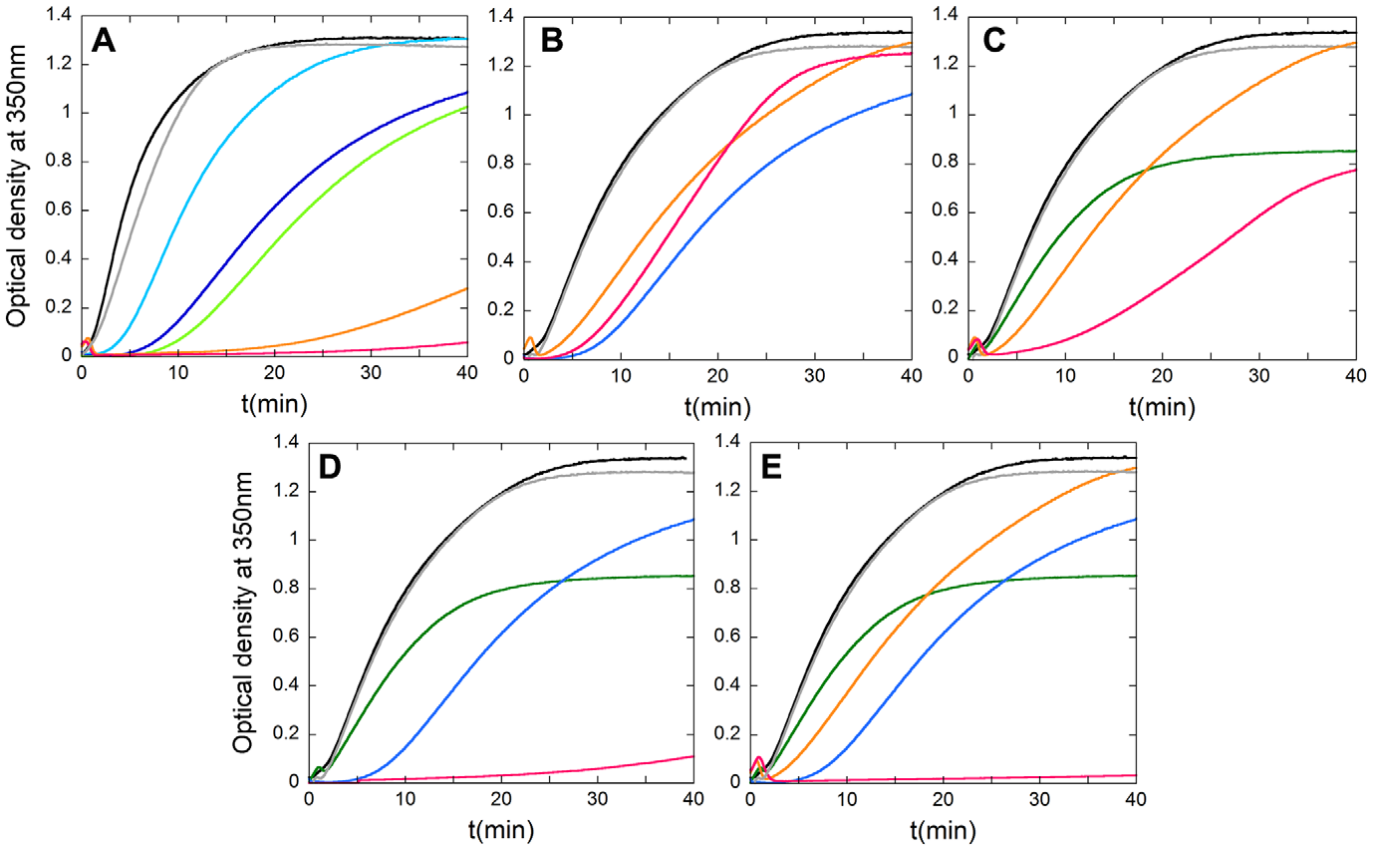
Inhibition of *in vitro* capsid assembly by cocktails of interfacial peptides. Reaction kinetics of CA polymerization using the conditions described in the legend for Fig. 3. (A) Inhibition by CAI under the macromolecular crowding conditions used for testing the inhibitory activity of the designed peptides described in this work. The traces are colored as follows: no peptide added (black); an inactive peptide at a peptide∶CA molar ratio of 10 (grey); CAI added at peptide∶CA ratios of 0.1 (light blue), 0.2 (blue), 0.3 (green), 0.4 (orange) or 0.5 (pink). (B) Inhibition by a cocktail of CAI and H8. No peptide added (black); an inactive peptide at a peptide∶CA ratio of 10 (grey); CAI added alone at a peptide∶molar ratio of 0.2 (blue); H8 added alone at a peptide∶CA molar ratio of 5 (orange); a cocktail of CAI and H8 added at the same molar ratios used for each peptide alone (pink). (C) Inhibition by a cocktail of CAC1M and H8. No peptide added (black); an inactive peptide at a peptide∶CA ratio of 10 (grey); H8 added alone at a peptide∶molar ratio of 5 (orange); CAC1M added alone at a peptide∶CA molar ratio of 2 (green); a cocktail of H8 and CAC1M added at the same molar ratios used for each peptide alone (pink). (D) Inhibition by a cocktail of CAC1M and CAI. No peptide added (black); an inactive peptide at a peptide∶CA ratio of 10 (grey); CAI added alone at a peptide∶molar ratio of 0.2 (blue); CAC1M added alone at a peptide∶CA molar ratio of 2 (green); a cocktail of CAI and CAC1M added at the same molar ratios used for each peptide alone (pink). (E) Inhibition by a cocktail of three peptides: CAC1M, CAI and H8. No peptide added (black); an inactive peptide at a peptide∶CA molar ratio of 10 (grey); CAI added alone at a peptide∶molar ratio of 0.2 (blue); CAC1M added alone at a peptide∶CA molar ratio of 2 (green); H8 added alone at a peptide∶molar ratio of 5 (orange); a cocktail of CAI, CAC1M and H8 added at the same molar ratios used for each peptide alone (pink).

Peptides CAI and its conformationally restricted derivative NYAD-1 had been previously shown to be efficient *in vitro* inhibitors of mature HIV-1 capsid assembly [17]. CAI was also very efficient at inhibiting *in vitro* capsid assembly under macromolecular crowding conditions (Fig. 5A), although its inhibitory activity was reduced in these conditions [53]. As the binding sites in CA of CAI/NYAD-1, CAC1/CAC1C/CAC1M, and H8 are different, we considered the use of cocktails of some of these interfacial peptides to inhibit HIV-1 capsid assembly at least as efficiently as with single peptides, but using lower doses of each peptide. In a putative *in vivo* application, such an approach could reduce secondary effects of individual peptides as well as the probability of virus escape.

CAI and NYAD-1 have essentially the same sequence and bind to the same site in CTD with a similar affinity, so we considered one of them would be enough for inclusion in our experiments as a reference and analysis of combined effects. We chose CAI because, unlike NYAD-1 and like the other peptides in our study, CAI is readily prepared, non-conformationally restricted and intrinsically unable to penetrate cells. Among the CAC1 peptides we chose CAC1M because of its higher solubility and affinity for CTD (Table 1). Cocktails of sub-inhibitory doses of peptides CAC1M, H8 and/or CAI were used in CA polymerization assays (Fig. 5). Although the individual peptides can achieve efficient assembly inhibition at higher doses (see above), at the low concentrations chosen for the new set of assays they caused only a minor inhibitory effect on capsid assembly. In these conditions, a mixture of peptides CAI+H8 was no better inhibitor than either peptide alone (Fig. 5B). In contrast, a mixture of peptides CAC1M+H8 led to an additive inhibitory effect (Fig. 5C). Remarkably, capsid assembly was essentially abolished when mixtures of peptides CAC1M+CAI (Fig. 5D), or CAC1M+H8+CAI (Fig. 5E) at those same low concentrations of each peptide were used. The three-peptide cocktail prevented the formation of even the slightest traces of assembled particles. The results revealed a functional exclusion between H8 and CAI, an additive effect between CAC1M and H8, and even a synergy between CAI and CAC1M, regarding the inhibitory activity of these peptides on the *in vitro* assembly of the mature HIV-1 capsid.

### Inhibition of HIV-1 infectivity by interfacial peptides

The structural study described here has shown that the designed peptides, alone or in combination, can efficiently inhibit the *in vitro* assembly of viral particles with the organization of authentic mature HIV-1 capsid, validating the successful design of interfacial inhibitors of capsid assembly. A full characterization of the inhibitory activity of these peptides in the more complex environment of infected cells will of course require an additional investigation using different, virological techniques. However, as a final part of our present study, and as a preliminary experiment for future *ex vivo* studies, we have tested whether the designed peptides could act as experimental antiviral agents by inhibiting HIV-1 infection of susceptible cultured cells. In order to do so, the peptides were first transported into cells.

#### Transport of peptides into cells susceptible to HIV-1 infection, and analysis of cytotoxicity

**Figure 6:**
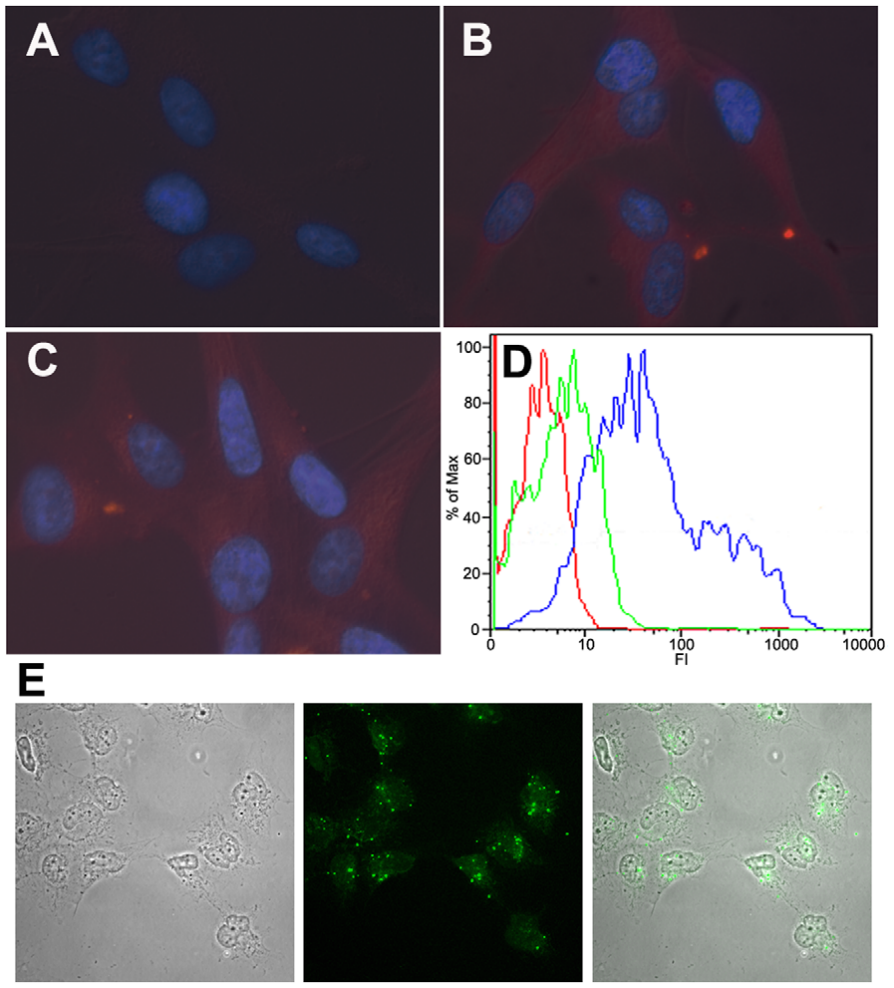
Transport of assembly inhibitor peptides into cells followed by fluorescence microscopy and flow cytometry. (A, B, C) Representative fluorescence microscopy images of U87-CD4-CXCR4 cells incubated for 3 h at 37°C with with no peptide added (A), or with 10 µg of Rhodamine-labelled CAI (B) or CAC1M (C) in complex with Chariot at a 1∶1 molar ratio. The cell nuclei are stained blue using DAPI; the presence of rhodamine-peptide conjugates is indicated by red fluorescence emission. (D) Flow cytometry analysis of U87-CD4-CXCR4 incubated for 3 h at 37°C with no peptide added (stained with propidium iodide) (red line), 50 µg of FITC-labelled CAI in the absence (green line) or in complex with Chariot at a 1∶1 molar ratio (blue line). (E) Representative fluorescence confocal microscopy images of peptide-transfected cells. NB324K cells were incubated for 3 h at 37°C with 10 µg of FITC-labelled CAI in complex with Chariot (Chariot∶peptide ratio of 1∶1). Left, difference interference contrast image showing the equatorial plane of several cells; center, green fluorescence emission of the same field and focal plane shown at left; right, superimposition of the two previous images, showing the presence of FITC-peptide conjugate inside the cells.

Use of a cell-penetrating heterologous peptide had allowed transport of CAI into cells [65], and we employed the commercial Chariot peptide to follow a similar approach. The conditions were defined using mainly HIV-1-susceptible U87-CD4-CXCR4 cells. Due to the high autofluorescence in these cells observed in the green channel, rhodamine-labelled peptides were used. To confirm that peptides with very different sequences could be transported, peptides CAC1M, CAI and H2 were tested in these assays. The fluorescence results (Figs. 6A, 6B and 6C, and results not shown) provided the first evidence that Chariot-mediated transport of peptides into HIV-1-susceptible cells had been achieved. Additional evidence for transport of peptides into U87-CD4-CXCR4 cells was provided by using FITC-labelled CAI in flow cytometry assays (Fig. 6D). Finally, we used confocal fluorescence microscopy to confirm that the peptides were not merely bound to cells, but actually internalized (Fig. 6E).

#### Inhibition of HIV-1 infectivity by peptides

We have tested the antiviral action of CAC1, CAC1M and H8. CAI (contrary to its conformationally restricted derivative NYAD-1) had previously failed to inhibit HIV-1 infection *ex vivo* as this peptide is unable to penetrate cells [17]. Thus, as we had done in the polymerization assays (see above), we included in the infectivity assays CAI in addition to the designed peptides. Also, CAI was somewhat more efficient than CAC1 and CAC1M and substantially more efficient than H8 at inhibiting *in vitro* mature capsid assembly, and this provided a reference for comparison of their inhibition of HIV-1 infection.

**Figure 7:**
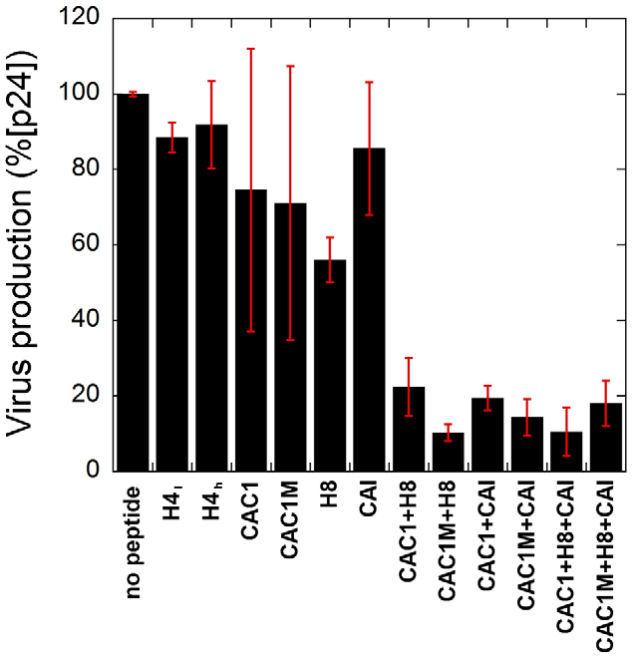
*Ex vivo* inhibition of HIV-1 infection by assembly inhibitor peptides. Peptides H4 (as a negative control), CAC1, CAC1M, H8, CAI and the indicated peptide cocktails were incubated with Chariot and transported into cells, and the cells were infected with HIV-1 strain HXB2 (0.0015 TCID50/cell) as described in Methods. The concentrations of peptides used in the experiments shown were: H4, 0.8 mM (labelled H4_l_) and 1.7 mM (labelled H4_h_); CAC1, CAC1M, CAI (alone or as part of a cocktail), 170 µM; H8 (alone or as part of a cocktail), 2.6 mM. Supernatants were collected at 72 h after infection, and the amount of CA produced (p24 antigen) was determined as described in Materials and Methods. The values obtained were normalized to the amount of p24 obtained in the absence of any peptide added. 100% virus production corresponds to 37,000 pg/ml of p24 antigen. The average and standard error from equivalent data obtained in several experiments are indicated.

The inhibitory activity of the peptides on HIV-1 infection was tested using cultured U87-CD4-CXCR4 cells. The concentrations of peptides used were not toxic to the cells. Cells were incubated with a mixture of the peptides (alone or in combination) and the Chariot peptide, and subsequently infected with HIV-1 strain HXB2 at a multiplicity of infection of 0.0015. The amount of progeny virus produced at 72 h post-infection was determined by titrating the amount of CA (p24 antigen) produced. Representative results are shown in Fig. 7. In the absence of added peptides, the infected cells produced considerable amounts of virus (yielding on average 37,000 pg/ml of p24 antigen). As expected, a negative control peptide (H4) that was unable to inhibit HIV-1 capsid assembly *in vitro*, was also unable to inhibit HIV-1 production *ex vivo*, even at peptide concentrations close to 2 mM.

Peptides CAC1, CAC1M, H8 and CAI alone, that did efficiently inhibit HIV-1 capsid assembly *in vitro*, showed at most a weak inhibitory activity on HIV-1 production *ex vivo* that required high (>1 mM) concentrations (Fig. 7). As these same purified peptides were very active at inhibiting CA polymerization, their poor individual effect on the inhibition of HIV-1 infection does not seem to be related mainly to a weak potency, or to a minor component of the peptide preparation being the only active species. Rather, Chariot-mediated transport of these peptides was an inefficient process and, in addition, short peptides are easily degraded inside cells. Thus, the poor inhibitory activity of HIV-1 infection by these peptides could be due to their very low concentration inside the infected cells.

Most remarkably, in spite of their inefficient transport and any degradation inside the cell, mixtures of CAC1/CAC1M+H8; CAC1/CAC1M+CAI; and CAC1/CAC1M+H8+CAI, substantially inhibited HIV-1 production (by about 90%) at the doses tested (Fig. 7), acting as antiviral agents *ex vivo*. These results are consistent with the high inhibitory activity of these mixtures on capsid assembly *in vitro*.

## Discussion

### Peptide CAC1 as an efficient assembly inhibitor

It may seem surprising that unconstrained, unstructured peptides like CAC1 and derivatives, that represent only a part of the CTD dimerization interface, are able to bind CTD and inhibit assembly of mature HIV-1 capsid-like particles almost as efficiently as the full-length, folded CTD itself. Part of the explanation may be related to the unusual features of the CTD-CTD interface. This interface resembles a typical high-affinity interface in buried surface area, number of residues, presence of a hydrophobic core [32], [33], and in containing as many as 9 residues per monomer that individually contribute as much as ≥6 kcal/mol to the dimerization reaction [39]. However, contrary to other structurally similar oligomerization interfaces [66], the CTD-CTD interface has a remarkably low affinity. Steric constraints imposed by the geometry of the capsid lattice [38] may contribute to capsid instability by distorting the minimum free energy, unconstrained conformation of the dimerization interface as present in the isolated CTD dimer. However, even when unconstrained in solution, CTD dimerization is a low-affinity reaction (*K_d_* about 10 µM) [32]. This intrinsically low affinity can be traced to i) the presence of interfacial residues that are involved in coulombic repulsions and other unfavorable interactions between CTD monomers [39], [67]; and ii) the very low thermodynamic stability of the monomeric form of CTD [62], which may underlie its unusually high structural plasticity in solution (e.g. [43], [44], [68]), on ligand binding (e.g. [16], [20], [69]), and during immature and mature capsid assembly (reviewed in refs. [3], [4]; see also ref. [70]). Specifically, in the CTD monomer helix 9 appears to be only partially and/or transiently folded [43], [44], and this helix must be structured during dimerization. Thus, the success of the helix 9-mimic CAC1 peptide and derivatives as interfacial inhibitors of HIV-1 capsid assembly may be mainly due to the fact that a similarly high entropic penalty must be paid for CTD-CAC1 association than for CTD-CTD association: neither the CAC1 peptide nor helix 9 in CTD are fully structured before dimerization occurs [43].

Even if those entropic effects are taken into account, it is still a fact that CAC1 includes most but not all of the CTD residues energetically important for CTD dimerization [39]. Thus, if bound CAC1 faithfully mimicked the conformation of helix 9 as part of CTD, a difference in true binding affinity larger than the one experimentally obtained could be expected (see the Results section). In fact, our mapping of the binding site in CTD of CAC1 revealed a substantial overlap with the CTD dimerization epitope, but the match between the two regions is far from perfect (compare Figs. 2B and 2C). In addition, other residues generally located close, but not at the CTD dimerization interface, were also affected by CAC1 binding. The very similar binding sites delineated for CAC1 and its derivatives CAC1C and CAC1M, and their potent inhibitory activity, further supports the validity of our mapping results.

A detailed interpretation of the observed structural and energetic differences and similarities between the CTD dimerization interface and the CTD-CAC1 interface must await the elucidation of the three-dimensional structure of the CTD-CAC1 complex. In the meantime, we tentatively propose that CAC1, despite containing the complete helix 9 sequence, may adopt on binding to CTD a folded conformation that resembles, but not fully mimics, that of helix 9 in dimeric CTD, while providing enough additional contacts for preserving a relatively strong binding to CTD. Despite the differences in binding and conformation, the overlap would be enough for the CTD-bound peptide to sterically impair CTD dimerization and CA polymerization.

### CAC1-derived peptides

Our rational design to increase CAC1 solubility and/or CTD binding affinity led in two steps to CAC1M, a CAC1-derived peptide that includes multiple amino acid substitutions and short N- and C-terminal extensions. These modifications were intended to disrupt a predicted aggregation-prone sequence, increase solubility, increase helical propensity and/or eliminate side chains that, in the complete CTD domain, restricted dimerization affinity. The design was moderately successful. In particular, substitutions S178A and/or Q192A may have contributed to some increase in the binding affinity and inhibitory potency of the peptide as intended. However, the affinity increase was smaller than when the same substitutions were introduced in the full-length CTD [39]. Again, this difference could be explained by the fact that, on binding CTD, CAC1 does not fully reproduce the conformation of helix 9 in the CTD domain.

### Peptide H8

A novel CA-derived peptide, H8, that was designed following a rationale similar to the one followed for CAC1 (the mimicking of interfacial helices), was also able to inhibit capsid assembly and HIV-1 infection, which tends to validate this approach for the design of inhibitor peptides. By comparison with the CAC1 results, it may be reasonable to propose that H8, which represents the sequence of helix 8 in CTD at the CTD-NTD interface, may bind NTD and competitively inhibit CTD-NTD heterodimerization during assembly of the mature capsid. Peptide H8 is not as active as CAC1 as an inhibitor of capsid assembly, probably because helix 8 (unlike helix 9), is fully formed in the CTD monomer, while peptide H8 in solution (like CAC1 and most short linear peptides) is not structured. Thus, H8-CA affinity may be lower than CTD-NTD affinity partly because of the high entropic cost of structuring this peptide.

### Cocktails of peptide inhibitors

The rationally designed peptides CAC1, its improved derivative CAC1M, and H8 add to the combinatorially selected peptide CAI [17] and its improved derivative NYAD-1 [18] as peptide inhibitors of HIV-1 capsid assembly *in vitro* and HIV-1 infectivity *ex vivo*. CAC1/CAC1M and H8 expand the choices of potential lead compounds for the design of peptidomimetics or other small molecule inhibitors of HIV-1 assembly. In addition, the unprecedented availability of several different peptide interfacial inhibitors allowed us to analyze the inhibitory activity of cocktails of these inhibitors on the *in vitro* assembly of the mature HIV-1 capsid.

The experiments revealed a negative interference between H8 and CAI, an additive effect between CAC1M and H8, and a synergistic effect between CAI and CAC1M. A tentative structural explanation for these observations can be proposed based on the different binding sites of these peptides on the CA molecule. CAI binds CTD at a region that involves parts of CA helices 8 and 9 [20], [21]. This region is close to the CTD-CTD interface, and clearly overlaps with the CTD-NTD interface. Thus, CAI binding may sterically prevent CTD-NTD heterodimerization, and it may also allosterically alter the CTD dimerization interface, without preventing dimer formation in solution but impairing the native quaternary organization of CA in the capsid lattice [17], [20], [21]. In full-length CA, binding of CAI to CTD and of H8 to NTD could sterically interfere with CTD-NTD association during capsid assembly, thus explaining the negative interference of these two peptides. In contrast, CAI and CAC1/CAC1M bind to non-overlapping patches in CTD, which would explain the observed additivity when mixtures of these peptides were used. The conformational rearrangement induced in CTD by the binding of CAI could even potentiate the binding of CAC1/CAC1M (and/or viceversa), leading to the observed synergistic inhibitory effects of these two peptides on capsid assembly. In analogy with current combination therapies, this observation provides initial support on the possibility of using cocktails of interfacial assembly inhibitors instead of a single assembly inhibitor, however potent, to reduce the emergence of resistant virus variants, which would need to accumulate mutations at different oligomerization interfaces.

### Inhibition of virus infection ex vivo

The present study has contemplated the rational design or modification, structural analysis and evaluation of peptides as inhibitors of HIV-1 capsid assembly using biophysical techniques. However, to provide an initial proof on their antiviral activity, we carried out some experiments regarding their assisted transport into cultured cells, and their inhibitory action on HIV-1 infection.

Similarly to that observed in *in vitro* assembly experiments, a mixture of peptides H8 and CAI was no better inhibitor of HIV-1 infection than each peptide alone, while a mixture of CAC1/CAC1M plus either H8 or CAI showed a substantial inhibition of virus infection. This observation provides a first suggestion that the antiviral activity of these peptides *ex vivo* is related, as expected from the *in vitro* results, with inhibition of mature HIV-1 capsid assembly. It could be argued that unless the peptides are incorporated in high enough concentrations within the budding virion, inhibition of mature capsid assembly inside the detached, maturing virus particle would not take place. However, it may be worth noting at this stage that the CTD-CTD and NTD-CTD interfaces observed in the mature capsid may not participate in immature capsid assembly [3], [4]. Thus, Gag molecules forming the immature capsid could bind substantial quantities of CAC1, CAC1M and H8 peptides to their sterically unimpeded binding sites in CA, thus locally increasing peptide concentration inside the budding virion.

It must be emphasized, however, that the present study has focused on the conceptual and experimental framework that goes from the structure-based design and modification of assembly-inhibition peptides to their structural characterization and evaluation in a defined, controlled, previously validated *in vitro* system that provides a simplified model of mature HIV-1 capsid assembly. It is not intended as a virological study in the complete mechanism of action of these peptides in the more complex environment inside HIV-1 infected cells, which will require a full set of different approaches and techniques.

To conclude, we have shown that rationally designed and improved peptides CAC1, CAC1M and H8 mimicking the sequence of protein helices involved in assembly of the mature HIV-1 capsid can act as efficient interfacial inhibitors of capsid assembly *in vitro* and have antiviral activity *ex vivo*.

## Supporting Information

Figure S1 **A representative row of the ^15^N-^1^H HSQC spectra of CTDW184A in the absence (black) or in the presence (blue) of CAC1M.** In the presence of peptide, the signal for Arg154 shows a decrease in intensity while the signal for Tyr164 and Phe168 remain unchanged. Data were acquired at 25°C.
(TIF)

Figure S2 **Analysis of self-association of peptides CAC1C (A) and CAC1M (B) by far-UV CD.** Traces indicate the fitting to 1∶1 stoichiometry. The ellipticity data were obtained at 222 nm (A) or 230 nm (B) and normalized. Data were acquired at 25°C.
(TIF)

Figure S3 **Thermal denaturation of CTD in the absence or presence of CAC1-derived peptides followed by far-UV CD.** Traces correspond to wild-type CTD in the absence of any peptide (black lines) or equimolar amounts of peptide (blue lines) CAC1C (A) or CAC1M (B). For convenience, the scales in (A) and (B) are different. The *T_m_*s obtained in the absence of peptides or in the presence of CAC1C were respectively 327.8±0.6 K and 325.0±2.0 K. The *T_m_* in the presence of CAC1M could not be accurately determined, but the different shape of the transition relative to absence of the peptide provided evidence of interaction.
(TIF)

Figure S4 **ITC analysis of CAC1 (A) or CAC1M (B) binding to wild-type CTD.** In each case, the titration curve is shown on top and the binding curve at the bottom. Experiments were carried out at 25°C.
(TIF)

Figure S5 **Transmission electron microscopy of CA assemblies obtained in polymerization assays.** Two magnifications are shown in (A) and (B). The tubular CA polymers observed present the same basic structural organization of authentic mature HIV-1 capsid-like structures with no pentameric “defects” [34].
(TIF)

Figure S6 ***In vitro*** **inhibition of HIV-1 capsid assembly by CAC1 (A), CAC1C (B) or CAC1M (C) in the absence of molecular crowding.** Reaction kinetics of CA polymerization was followed as indicated in Fig. 3. CA concentration was 20 µM and no crowding agent was added. The traces are colored as follows: no free peptides added (black); an inactive peptide at a peptide∶CA molar ratio of 10 (grey); CAC1 (A), CAC1C (B) or CAC1M (C) peptides added at inhibitor∶CA molar ratios of 0.2 (blue) or 0.5 (orange).
(TIF)

Table S1 Residues of CTDW184A whose signal intensity in the HSQC spectra changed upon peptide addition.
(DOC)
