# Supplementary figures and images for: Rationally Designed Interfacial Peptides Are Efficient In Vitro Inhibitors of HIV-1 Capsid Assembly with Antiviral Activity
Source: PLoS One. 2011 Sep 8;6(9):e23877. doi: 10.1371/journal.pone.0023877 (PMC3169566; doi:10.1371/journal.pone.0023877)

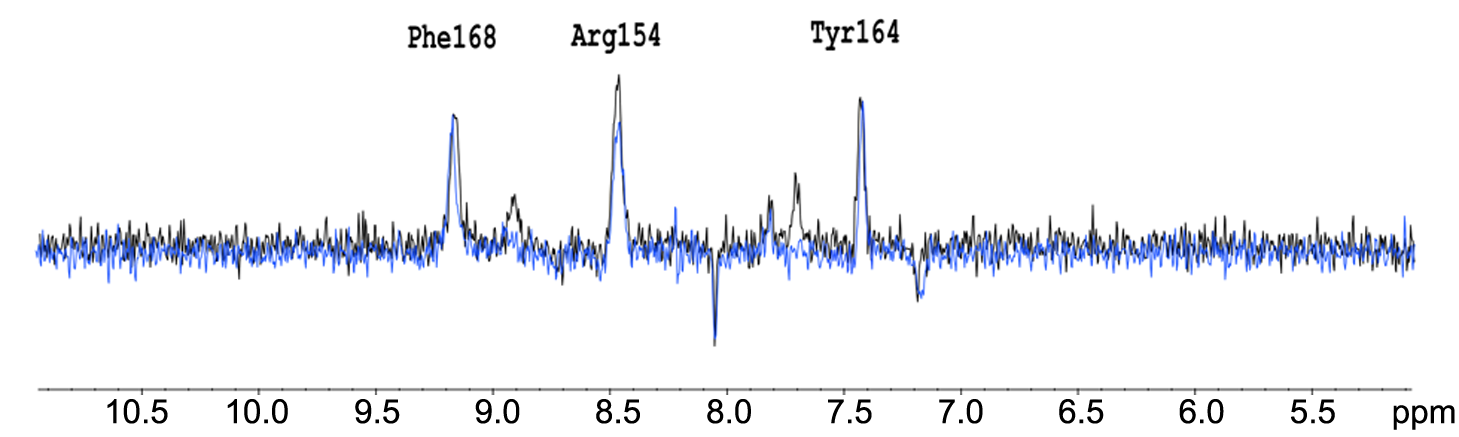

Supplement: Figure S1 — A representative row of the 15N-1H HSQC spectra of CTDW184A in the absence (black) or in the presence (blue) of CAC1M. In the presence of peptide, the signal for Arg154 shows a decrease in intensity while the signal for Tyr164 and Phe168 remain unchanged. Data were acquired at 25°C. (TIF) [file pone.0023877.s001.tif]

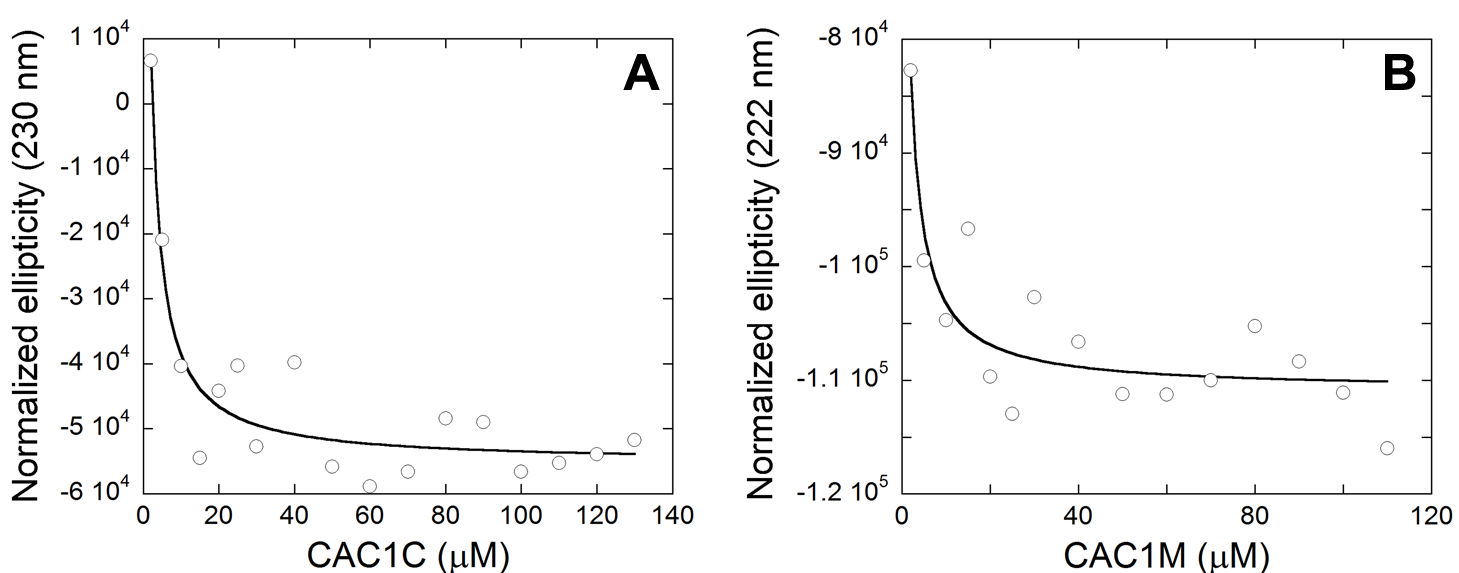

Supplement: Figure S2 — Analysis of self-association of peptides CAC1C (A) and CAC1M (B) by far-UV CD. Traces indicate the fitting to 1∶1 stoichiometry. The ellipticity data were obtained at 222 nm (A) or 230 nm (B) and normalized. Data were acquired at 25°C. (TIF) [file pone.0023877.s002.tif]

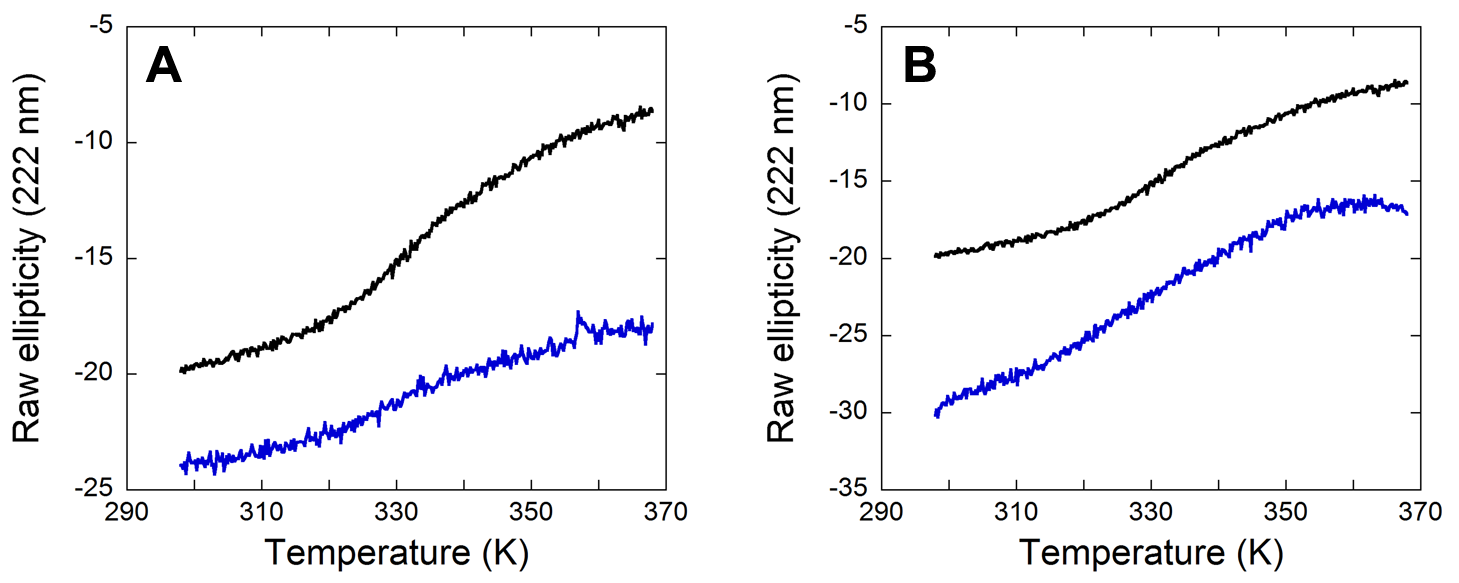

Supplement: Figure S3 — Thermal denaturation of CTD in the absence or presence of CAC1-derived peptides followed by far-UV CD. Traces correspond to wild-type CTD in the absence of any peptide (black lines) or equimolar amounts of peptide (blue lines) CAC1C (A) or CAC1M (B). For convenience, the scales in (A) and (B) are different. The Tms obtained in the absence of peptides or in the presence of CAC1C were respectively 327.8±0.6 K and 325.0±2.0 K. The Tm in the presence of CAC1M could not be accurately determined, but the different shape of the transition relative to absence of the peptide provided evidence of interaction. (TIF) [file pone.0023877.s003.tif]

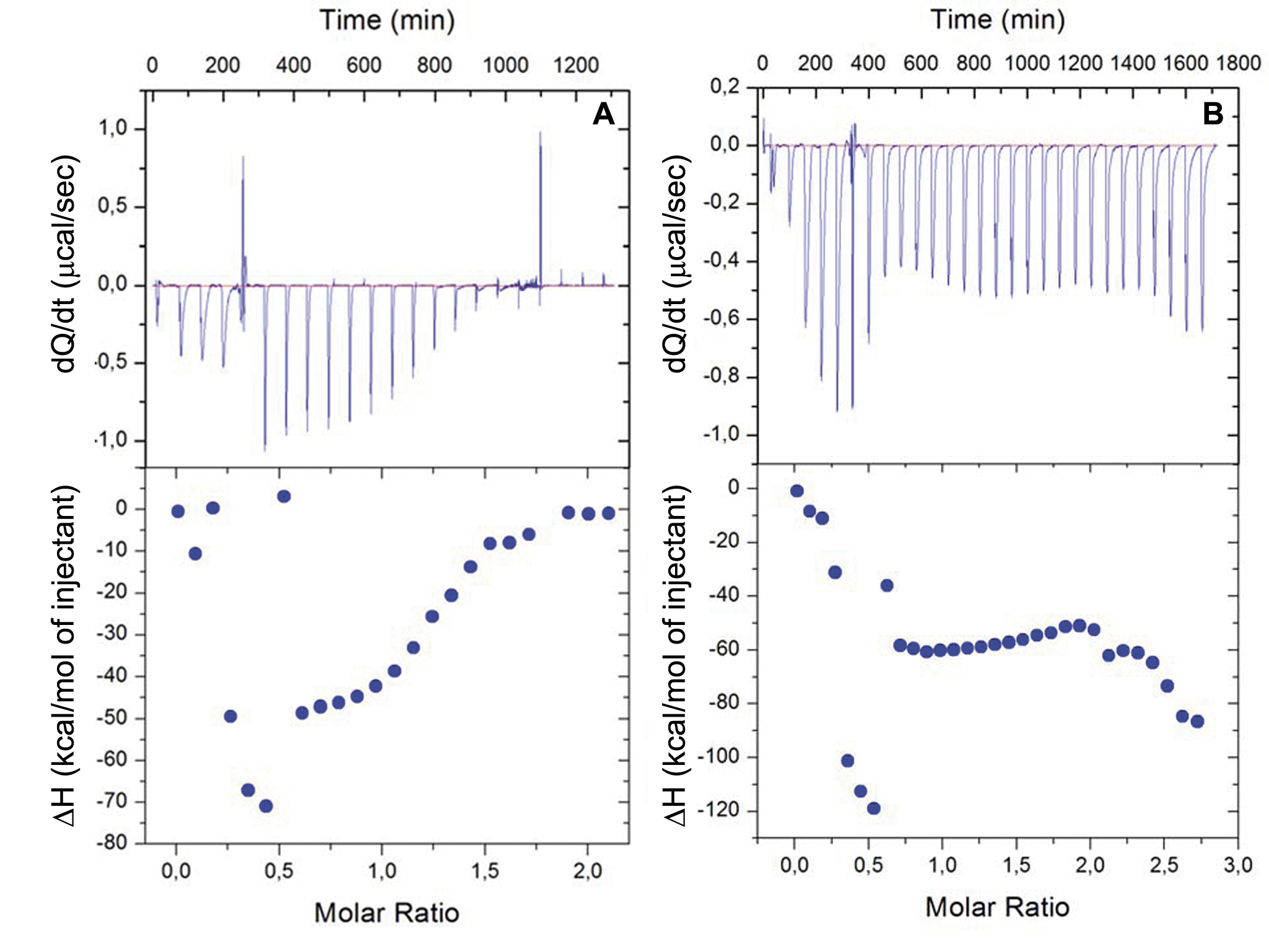

Supplement: Figure S4 — ITC analysis of CAC1 (A) or CAC1M (B) binding to wild-type CTD. In each case, the titration curve is shown on top and the binding curve at the bottom. Experiments were carried out at 25°C. (TIF) [file pone.0023877.s004.tif]

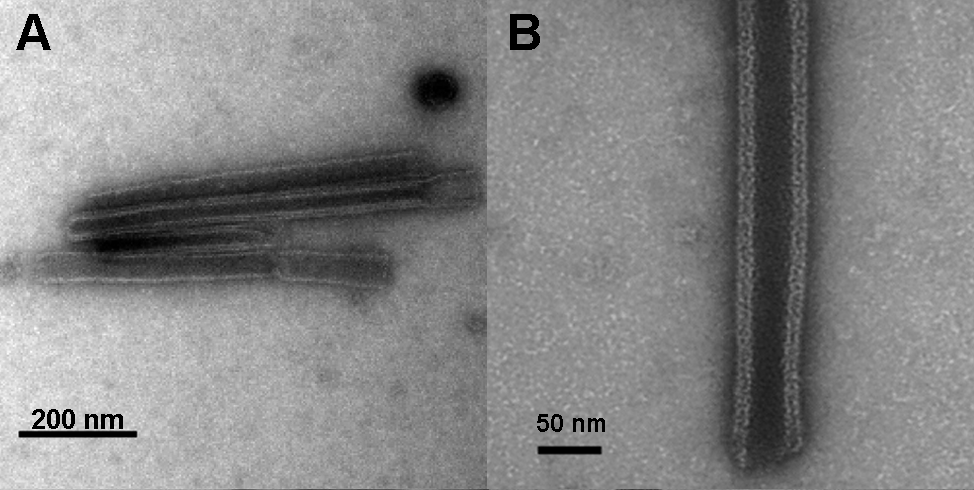

Supplement: Figure S5 — Transmission electron microscopy of CA assemblies obtained in polymerization assays. Two magnifications are shown in (A) and (B). The tubular CA polymers observed present the same basic structural organization of authentic mature HIV-1 capsid-like structures with no pentameric “defects” [34]. (TIF) [file pone.0023877.s005.tif]

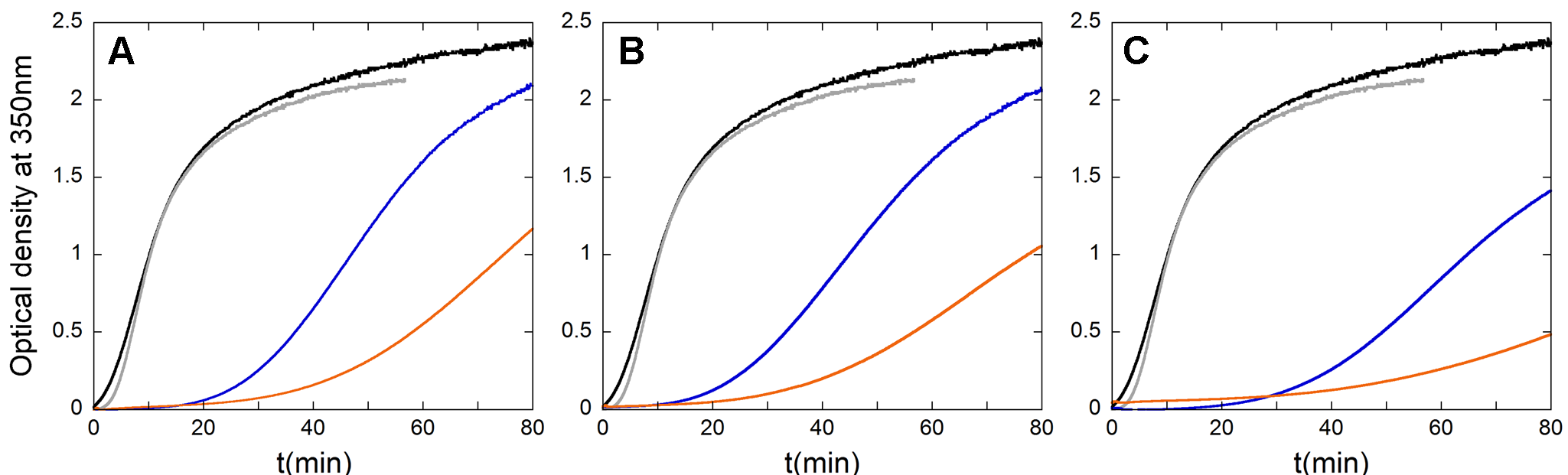

Supplement: Figure S6 — In vitro inhibition of HIV-1 capsid assembly by CAC1 (A), CAC1C (B) or CAC1M (C) in the absence of molecular crowding. Reaction kinetics of CA polymerization was followed as indicated in Fig. 3. CA concentration was 20 µM and no crowding agent was added. The traces are colored as follows: no free peptides added (black); an inactive peptide at a peptide∶CA molar ratio of 10 (grey); CAC1 (A), CAC1C (B) or CAC1M (C) peptides added at inhibitor∶CA molar ratios of 0.2 (blue) or 0.5 (orange). (TIF) [file pone.0023877.s006.tif]
