# Supplementary material for: Rationally Designed Interfacial Peptides Are Efficient In Vitro Inhibitors of HIV-1 Capsid Assembly with Antiviral Activity
Source: PLoS One. 2011 Sep 8;6(9):e23877. doi: 10.1371/journal.pone.0023877 (PMC3169566; doi:10.1371/journal.pone.0023877)
Supplement: Table S1 — Residues of CTDW184A whose signal intensity in the HSQC spectra changed upon peptide addition. (DOC) [file pone.0023877.s007.doc]

**Table S1.** Residues of CTDW184A whose signal intensity in the HSQC spectra changed upon peptide addition.

| Protein region | Residue^a^ | Variation in signal intensity^a^ | | |
| --- | --- | --- | --- | --- |
|  |  | +CAC1 | +CAC1C | +CAC1M |
| N-terminus | I150 | -17 | -17 | -17 |
| MHR (152-172) and Helix 8 (160-172) | R154 | -19 | -19 | -19 |
|  | Q155 |  |  | -10 |
|  | V165 | -10 |  |  |
|  | R167 | -14 | -14 | -14 |
|  | K168 |  | -12 |  |
| H8-H9 loop | Q176 |  | -10 |  |
|  | R173 |  |  | -11 |
| Helix 9 (178-191) | Q179 |  | -11 | -11 |
|  | E180 | -16^b^ | -16^b^ |  |
|  | V181 |  | -12 |  |
|  | N183 |  |  | -10 |
|  | A184 | -11 | -11 | -11 |
|  | M185 | -16 | -16 | -16 |
|  | T188 | -19 | -19 | -19 |
|  | L190 | -17^b^ | -17^b^ | -17^b^ |
|  | V191 |  | -18 | -18 |
| H9-H10 loop | N193 | -21^b^ |  |  |
| Helix 10 (195-202) | D197 | -17 | -17 | -17 |
|  | C198 | -21 |  | -21 |
|  | K199 | -12 |  |  |
|  | T200 | -19 | -19 | -19 |
|  | I201 |  |  | -12 |
|  | L202 | -10 |  | -10 |
| H10-H11 loop | K203 | -14 | -14 | -14 |
|  | A204 | -10 |  | -10 |
|  | G206 |  | -10 |  |
|  | A208 | -11 |  |  |
| Helix 11 (209-214) | A209 | -11 | -11 |  |
|  | T210 | -21 |  |  |
|  | L211 |  | -15 | -15 |
|  | E212 | -12 | -12 |  |
|  | M214 |  | -18 | -18 |
| C-terminus | M215 |  |  | -11 |
|  | A217 | -18 | -18 | -18 |
|  | C218 |  |  | -14 |
|  | Q219 | -16 | -16 | -13 |
|  | V221 | -10 | -10 |  |

^a^The variations (decrease) in signal intensity (arbitrary units) are normalized to that of the C-terminal residue Leu231. The CTD residues listed are those that showed a change in normalized intensity of the corresponding cross-peak larger than 10 units when the CTDW184A spectra in the absence ot presence of a peptide (CAC1, CAC1C or CAC1M) were compared. See Fig.S1 for one example including the row corresponding to the ^15^N resonance of Arg154.

^b^ Signal for Glu180 overlaps with those of Ser149 and His226; that of Leu190 with Leu151; and that of Asn193 with Glu178.
